# Psychiatry in a Time of Crisis: Paving the Road to Recovery—A Commentary by the Canadian Academy for Addiction Psychiatry (CAAP)

**DOI:** 10.1177/07067437251389845

**Published:** 2025-10-28

**Authors:** Reinhard Michael Krausz, Robert L. Tanguay, Martha J. Ignaszewski, Valerie Primeau, Vijay Seethapathy

**Affiliations:** 1 Department of Psychiatry, 12358 University of British Columbia, Vancouver, BC, Canada; 2 Department of Psychiatry and Surgery, Cumming School of Medicine, 70401 University of Calgary, AB, Canada; 3 Hotchkiss Brain Institute, University of Calgary, AB, Canada; 4 Mathison Centre for Mental Health Research and Education, University of Calgary, AB, Canada; 5 Substance Use and Concurrent Disorders, Department of Child and Adolescent Psychiatry, 37210 BC Children's Hospital, Vancouver, BC, Canada; 6 Department of Psychiatry, Complex Pain and Addiction Services, 8167 Vancouver General Hospital, Vancouver, BC, Canada; 7 Department of Psychiatry, 27364 North Bay Regional Health Centre, North Bay, ON, Canada; 8 60299 BC Mental Health & Substance Use Services, Burnaby, BC, Canada

**Keywords:** opioid overdose crisis, mental health and substance use, addiction psychiatry, evidence-based training, person-centred care, substance use treatment protocols, families and lived experience in substance use disorder care, implementation science

Substance use disorders represent a core chapter in psychiatric classification. The majority of patients in emergency settings aiming for inpatient care suffer from substance use and concurrent mental illness across diagnostic categories. This complexity is defining a different level of challenges for the system of care we need to prepare for in training, research and across the system of interventions and healthcare as a system.

Since 2014, North America has been in the midst of the most devastating public health crisis related to a mental health condition, with historic levels of mortality due to overdose fatalities.^1,2^ This crisis has starkly illuminated the existing limitations within our mental health and substance use care systems, including fragmented care pathways, a significant gap in service availability, a shortage of trained personnel, and a deficit in clinical research infrastructure and academic leadership. These weaknesses have hindered our ability to mount a successful and timely response to the opioid overdose epidemic.

Similar to how the psychiatric system of care has mobilized rapid public health responses during the COVID-19 pandemic and the HIV/AIDS epidemic, it must now implement a bold and systematic strategy to address the escalating mortality from opioid overdose and take responsibility for leading this response.^3^ Analogously, transdisciplinary collaboration across all medical and allied health disciplines is needed to establish an effective response to address a mortality rate, which is already contributing to a declining life expectancy across North America.^4^

Given the prevalence of concurrent mental health disorders among individuals with high-risk substance use,^5^ which in itself is a complex problem involving functional changes to the brain, in addition to the specific expertise in dealing with psychotropic substances and pharmacotherapy of mental health conditions—psychiatry needs to play a more effective and active role leading structural, transformational, and innovative change.

The Canadian Academy for Addiction Psychiatry (CAAP) is a new national professional organization that was established to support systematic change in high-risk substance use disorder and complex concurrent mental illness management and care. Focuses include contributing to capacity building and empowerment to address one of the biggest challenges in the mental healthcare system, represented in different focus areas.

**Pillar 1:** Substantial and evidence-based training starting at universities, of all professions involved, based and integrated with research, needs to be a foundation for an effective response to the crisis.

**Pillar 2**: Development of translational and substantial clinical research, especially in response to the current mortality crisis. The current situation is demanding, and adaptation of clinical protocols across all interventions is required. As in the response to the COVID-19 pandemic, the development of new treatment approaches and their implementation needs to be front and centre.

The work on improved local assessment of opioids in collaboration with chemistry and clinicians, the improvement of withdrawal management for high-potent opioids combining behavioural pharmacology and clinical research or the overall adaptation of treatment protocols in changing drug markets are some examples. A partnership with healthcare system research and implementation research will be essential to adapt services on a system level. We need to build capacity, and new methods are needed for that, including, for example, virtual care.

**Pillar 3**: Evidence-informed advocacy. These policies should be developed collaboratively by experts across healthcare, research, and decision-making bodies, grounded in the latest evidence and a commitment to improved outcomes. The current discussion on mandatory care for some of these patients and the necessary informed input is an example.

**Pillar 4**: Improved collaboration and a dialogue between disciplines to overcome fragmentation and unnecessary competition in order to improve outcomes. The inclusion of families, peers, and individuals with lived experience is essential, creating a person-focused and inclusive, multiperspective approach that is both evidence-driven and outcome-focused. Complex concurrent disorders in this field are a significant and growing clinical challenge, which requires attention in the overall curricula and specialized training. We want to help especially psychiatric teams across the clinical trajectories, improving care for this population. Beyond the efforts in primary care and addiction medicine, we want to help overcome access barriers and address integrated care of mental illness, trauma and high-risk substance use, including a strategy for the current opioid overdose mortality. We see this as a priority population, which is underserved and needs better evidence-based pharmacological and psychosocial interventions. The initiative is advocating for a new model of integrated care, removing barriers and addressing stigma in the healthcare system. This includes initiatives for more clinical research capacities and the building of competitive networks in this field.

In big professional network organizations, “Addiction Psychiatry” didn’t get the attention and support it would need to play the role it should play in the development of the mental healthcare system. Our model builds on the positive experiences, for example, in internal medicine, combining high levels of specialized services with the overall development of the discipline.

The important work in general addiction medicine is addressing important domains from a service delivery perspective. We want the expertise of addiction psychiatry to add the high-quality specialized treatment of concurrent mental disorders to the field.

## Between Science and Lived Experience

The inaugural conference 2024 of the newly founded CAAP was held on October 21^st^/22^nd^ in Vancouver, British Columbia, hosting over 200 participants. Featured panels included the challenges of the healthcare system in the current crisis, comparing experiences in Alberta and British Columbia, the controversy on treatment of alcohol dependent patients with concurrent depression, and the major challenge of trauma and substance use.

Sid Kennedy, the cofounder of the Canadian Network for Mood and Anxiety Treatments,^6^ one of the most successful mental health networks advocating for the improvement of care in mood disorders, summarized his impression of the conference as a mixture of good science and lived experience.

A lot of sensitive questions from mandated care of patients with substance use disorders to the public controversy on depression treatment of alcohol dependent individuals, or healthcare system prerequisites and structures to address the current opioid overdose crisis, were discussed with passion and common purpose.^7,8^ A promising start, the near future will show CAAP's potential on how it can contribute to the high aspirations of changing psychiatry and mental healthcare for the better, addressing the current public health crisis more effectively and bringing down historic mortality rates associated with the opioid crisis.

Change is necessary to address a historic challenge in our discipline and prepare for change. Taking on the responsibility for the current mortality crisis and other challenges with the substantial psychiatric tools across the field of complex concurrent mental disorders, including substance use disorders, is an important step. The CAAP provides the network and knowledge to support those who care especially for this population, redefining the focus of our discipline and making it better.
